# A WIN Consortium phase I study exploring avelumab, palbociclib, and axitinib in advanced non‐small cell lung cancer

**DOI:** 10.1002/cam4.4635

**Published:** 2022-03-20

**Authors:** Benjamin Solomon, Ana Callejo, Jair Bar, Guy Berchem, Lyudmila Bazhenova, Pierre Saintigny, Fanny Wunder, Jacques Raynaud, Nicolas Girard, J. Jack Lee, Raed Sulaiman, Bruce Prouse, Catherine Bresson, Hila Ventura, Shai Magidi, Eitan Rubin, Brandon Young, Amir Onn, Brian Leyland‐Jones, Richard L. Schilsky, Vladimir Lazar, Enriqueta Felip, Razelle Kurzrock

**Affiliations:** ^1^ Avera Cancer Institute Sioux Falls South Dakota USA; ^2^ Vall d'Hebron Hospital Campus and Institute of Oncology (VHIO) Barcelona Spain; ^3^ Chaim Sheba Medical Center Tel Hashomer Israel; ^4^ Centre Hospitalier de Luxembourg Luxembourg Institute of Health Luxembourg City Luxemburg; ^5^ University of California San Diego Moores Cancer Center San Diego California USA; ^6^ Centre Léon Bérard, Cancer Research Center of Lyon University of Lyon Lyon France; ^7^ Worldwide Innovative Network (WIN) Association ‐ WIN Consortium Villejuif France; ^8^ ARC Foundation for cancer research Villejuif France; ^9^ Institut Curie Paris France; ^10^ The University of Texas MD Anderson Cancer Center Houston Texas USA; ^11^ Ben‐Gurion University of the Negev Be'er Sheva Israel; ^12^ Bowden Laboratory Murrieta California USA

**Keywords:** anti‐PD‐L1, CDK4/6, genomics, NSCLC, phase I, transcriptomics, VEGFR

## Abstract

**Background:**

The Worldwide Innovative Network (WIN) Consortium has developed the Simplified Interventional Mapping System (SIMS) to better define the cancer molecular milieu based on genomics/transcriptomics from tumor and analogous normal tissue biopsies. SPRING is the first trial to assess a SIMS‐based tri‐therapy regimen in advanced non‐small cell lung cancer (NSCLC).

**Methods:**

Patients with advanced NSCLC (no *EGFR*, *ALK*, or *ROS1* alterations; PD‐L1 unrestricted; ≤2 prior therapy lines) received avelumab, axitinib, and palbociclib (3 + 3 dose escalation design).

**Results:**

Fifteen patients were treated (five centers, four countries): six at each of dose levels 1 (DL1) and DL2; three at DL3. The most common ≥Grade 3 adverse events were neutropenia, hypertension, and fatigue. The recommended Phase II dose (RP2D) was DL1: avelumab 10 mg/kg IV q2weeks, axitinib 3 mg po bid, and palbociclib 75 mg po daily (7 days off/21 days on). Four patients (27%) achieved a partial response (PR) (progression‐free survival [PFS]: 14, 24, 25 and 144+ weeks), including two after progression on pembrolizumab. Four patients attained stable disease (SD) that lasted ≥24 weeks: 24, 27, 29, and 64 weeks. At DL1 (RP2D), four of six patients (66%) achieved stable disease (SD) ≥6 months/PR (2 each). Responders included patients with no detectable PD‐L1 expression and low tumor mutational burden.

**Conclusions:**

Overall, eight of 15 patients (53%) achieved clinical benefit (SD ≥ 24 weeks/PR) on the avelumab, axitinib, and palbociclib combination. This triplet showed antitumor activity in NSCLC, including in tumors post‐pembrolizumab progression, and was active at the RP2D, which was well tolerated.

NCT03386929 clinicaltrial.gov

## Graphical abstract

The combination of anti‐PD‐L1 immunotherapy plus VEGFR/CDK4/6 targeting agents is well tolerated and active in patients with advanced NSCLC, even in those whose tumors progressed on prior checkpoint blockade.

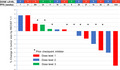

## 1 BACKGROUND

Non‐small cell lung cancer (NSCLC) is among the most prevalent and lethal malignancies worldwide, with approximately 2.1 million new cases and 1.8 million deaths per year.^1^ Most patients are diagnosed with locally advanced or metastatic disease at presentation, and relapses are common among patients initially presenting with localized disease.^2^ Although the advent of targeted therapies and immune oncology have improved outcomes in patients with advanced NSCLC, there are many patients who are not eligible for these treatments or who do not respond.

To this point, precision oncology approaches in NSCLC have largely focused on single driver alterations. This strategy has proven highly successful in cases of NSCLC with *EGFR* mutations and *ALK* fusions, among others.^3^, ^4^ However, single driver mutations are uncommon in the larger NSCLC population, and they are particularly uncommon in heavy smokers and patients with squamous cell carcinoma.^5^ More frequently, by next generation sequencing (NGS), NSCLC is found to harbor multiple active oncogenic drivers and silenced tumor suppressor genes, leading to a much more complex set of mechanisms that drive tumor growth.^6^

The Worldwide Innovative Network (WIN) for personalized cancer therapy is a non‐governmental, nonprofit organization with numerous academic and industry members. WIN was founded with the goal of simultaneously accelerating the pace and decreasing the cost of translational and clinical genomic‐based cancer research worldwide. WIN has hypothesized that, in order to more fully characterize the important and most actionable alterations in NSCLC, it is important to analyze both tumor genomics as well as transcriptomics, compared to that of analogous normal tissue for the latter. This approach has been successfully implemented in a previously published WIN trial––WINTHER.^7^, ^8^ Based upon this principle, WIN has developed the simplified interventional mapping system (SIMS) algorithm in order to predict treatment response to combination therapy with multiple‐targeted agents.^9^

The SPRING trial was developed to assess the safety and tolerability of a tri‐therapy‐targeted regimen combining the anti‐PD‐L1 agent avelumab, the antiangiogenic compound axitinib (vascular endothelial growth factor receptor [VEGFR] inhibitor), and the CDK4/6 inhibitor palbociclib. This combination was based on several lines of evidence: (i) VEGFR inhibitors are associated with immunomodulatory effects such as enhancing tumor infiltration of immune cells and attenuating immuno‐suppressive effects of myeloid‐derived suppressor cells^10^; (ii) antiangiogenic therapy can also improve anti‐PD1/L1 treatment by generating intra‐tumor high endothelial venules (HEVs) that facilitate enhanced cytotoxic T‐cell (CTL) infiltration, activity, and tumor cell destruction^11^; (iii) conversely, anti‐PD‐L1 therapy can sensitize tumors to antiangiogenic therapy and prolong its efficacy^11^; (iv) an immune checkpoint inhibitor together with axitinib was associated with improved outcomes in renal cell cancer^12^; and (v) novel mechanisms for regulating PD‐L1 protein stability utilize cyclin D‐CDK4 molecules.^13^ Finally, our previous study utilizing the simplified interventional point matching system (SIMS), based on transcriptomic/genomic analysis of matched tumor and normal biopsies, yielded the anti‐PD‐L1/anti‐VEGFR/‐CDK4/6 inhibitor triplet combination as possibly affecting a significant subset of patients with NSCLC (9).

Herein, we describe the results of the Phase I trial of the novel triplet of avelumab (anti‐PD‐L1 immunotherapy), axitinib (anti‐VEGFR), and palbociclib (CDK4/6 inhibitor) in NSCLC, which showed activity and tolerability in NSCLC.

## 2 METHODS

### 2.1 Aim and design of the study

The study was designed and conducted by the Worldwide Innovative Network (WIN) Consortium, an international collaboration of academic research institutions and industries aimed at advancing personalized cancer medicine. The SPRING trial is a Phase I, 3 + 3 dose escalation study that enrolled patients with advanced/metastatic NSCLC (NCT03386929). All patients provided informed consent according to local institutional review board (IRB) guidelines.

All patients were treated with avelumab, axitinib, and palbociclib according to the dose level enrolling at the time of study entry (Table S1). The primary objective of the study was to determine the safety of the three‐drug combination therapy and to derive recommended doses for further study. Secondary objectives were to assess activity parameters including response rate (RR) by RECIST 1.1,^14^ duration of response, progression‐free survival (PFS), and overall survival (OS). PFS was calculated from start of therapy until progression; for patients removed from study without progression, PFS was calculated from start of study until start of next treatment (or until progression, if progression documented before start of next treatment). Prior to starting treatment, all patients underwent biopsy of both tumor tissue and normal endobronchial mucosa, without any complications.

### 2.2 Participants' characteristics

Key inclusion criteria were as follows: Eastern Cooperative Oncology Group (ECOG) performance status of 0–1, ≤2 prior lines of therapy in the advanced/metastatic setting, adequate hematologic, hepatic, and renal function, and willingness to undergo radiology‐ guided needle biopsy of tumor tissue and biopsy of normal endobronchial mucosa by bronchoscopy. Patients with asymptomatic (treated or untreated) brain metastases were allowed. The following were key exclusion criteria: *EGFR* mutation, *ALK* fusion, *ROS1* fusion (if tested), and MET alteration (if tested). Patients requiring ongoing anticoagulation or with a bleeding diathesis were also excluded.

### 2.3 Interventions

Patients underwent radiology‐guided needle biopsy of tumor tissue and biopsy of normal endobronchial mucosa by bronchoscopy. The genomic analysis described was most commonly performed by Foundation Medicine Clinical Laboratory Improvement Amendment (CLIA) next generation sequencing (NGS) (236 genes) (foundationmedicine.com). The assay identifies all four classes of genomic alterations: base substitutions, deletions and insertions, copy number alterations, and rearrangements. Microsatellite status (a measure of microsatellite instability, or “MSI”) was determined by assessing indel characteristics at 114 homopolymer repeat loci in, or near, the targeted gene regions of the Foundation Medicine assay. The TMB categorization (into low, intermediate, and high) was assigned as follows: low = 0–5 mutations/mb; intermediate = 6–19 mutations/mb; high = ≥20 mutations/mb). TMB was defined as the number of somatic, coding, base substitution, and indel mutations per megabase of genome analyzed. Non‐coding and germline alterations were not counted. Alterations listed as known somatic alterations in COSMIC and truncations in tumor suppressor genes were not counted, since the assay genes are biased toward genes with functional mutations in cancer.

### 2.4 Treatment

Avelumab was administered at the study center by IV infusion with appropriate premedication every 2 weeks. Axitinib was taken by mouth twice per day. Palbociclib was taken orally once per day on days 8–28 of each 28‐day cycle (see Table S1 for dose levels and how the starting dose was determined). A clinical monitoring committee including all investigators met via weekly scheduled teleconference calls to monitor and direct management of any adverse events (AEs). AEs were monitored from the time of informed consent through the follow‐up period of 90 days after discontinuation of study treatment and were graded according to Common Terminology Criteria for Adverse Events (CTCAE) version 4.03.^15^ Causal relationship between study treatment and AEs was determined by the investigators and the clinical monitoring committee, and events were considered drug related if classified by the investigator as at least possibly related to study treatment.

#### 2.4.1 Maximum‐tolerated dose definition

If there is dose‐limiting toxicity (DLT) (≥grade 3 clinically relevant toxicity at least possibly related to drug) at a dose level in the first 4 weeks, three new patients were to be included at the same level. If a second patient has a DLT at that dose level, the dose level will be declared above the MTD and the next lower dose level will be expanded to six patients from three patients. The next lower dose level will be declared the MTD providing that no more than one patient of six has dose‐ limiting toxicity (or <one third of patients have DLTs).

## 3 RESULTS

### 3.1 Patient characteristics

Patients were enrolled at five separate sites worldwide: Avera Cancer Center, Sioux Falls, SD, USA; Moores Cancer Center, UCSD, La Jolla, CA, USA; Vall d'Hebron Institute of Oncology, Barcelona, Spain; Centre Hospitalier du Luxembourg, Luxembourg; Chaim Sheba Medical Center, Ramat Gan, Israel. A total of 15 patients were enrolled and treated with avelumab, axitinib, and palbociclib; five women and 10 were men. Median age was 67 years (range 51–80 years). One patient was treated in first line, nine patients in second line, and five patients in third line in the metastatic setting. The most common histology was adenocarcinoma. Ten patients had been previously treated with regimens containing immune checkpoint inhibitors. (Table 1).

**TABLE 1:** Patient characteristics

Characteristic	
Median age (years)	67 (range 51–80)
Sex (N)	
Male	10
Female	5
Prior lines of therapy in metastatic setting (*N*)	
None	1
1 line	9
2 lines	5
Number of patients who received prior checkpoint inhibitor	10
Histology	
Adenocarcinoma	10
Squamous undifferentiated cell carcinoma	3
Undifferentiated large cell carcinoma	1
Large cell neuroendocrine carcinoma	1
Number of patients by site	
Avera Cancer Institute, South Dakota, USA	5
VHIO, Barcelona, Spain	3
Chaim Sheba Medical Center, Tel Hashomer, Israel	3
University of California San Diego Moores Cancer Center, CA, USA	2
Centre Hospitalier du Luxembourg, Luxembourg	2

### 3.2 Side effects

The most common Grade 3 or higher adverse events that were at least possibly treatment related were neutropenia, hypertension, lymphopenia, and fatigue (Table 2). Other Grade 3 or higher adverse events included respiratory failure, leukopenia, diarrhea, infusion reaction, alanine aminotransferase increased, weight loss, hypertriglyceridemia, hyponatremia, thrombosis, and palmar‐plantar erythrodysesthesia. A single occurrence of Grade 5 respiratory failure was documented as possibly drug related. This event occurred in a patient with underlying cardiopulmonary comorbidities.

**TABLE 2:** Grade ≥ 3 adverse events at least possibly drug related

Adverse event	Number of patients (%)
*Respiratory failure*	
Grade 5	1 (7%)
*Neutropenia*	
Grade 4	1 (7%)
Grade 3	3 (20%)
*Lymphopenia*	
Grade 4	1 (7%)
Grade 3	2 (13%)
*Leukopenia*	
Grade 4	1 (7%)
*Diarrhea*	
Grade 3	1 (7%)
*Fatigue*	
Grade 3	2 (13%)
*Infusion reaction*	
Grade 3	1 (7%)
*Alanine aminotransferase increased*	
Grade 3	1 (7%)
*Weight loss*	
Grade 3	1 (7%)
*Hypertriglyceridemia*	
Grade 3	1 (7%)
*Hyponatremia*	
Grade 3	1 (7%)
*Thromboembolic event*	
Grade 3	1 (7%)
*Palmar‐plantar erythrodysesthesia*	
Grade 3	1 (7%)
*Hypertension*	
Grade 3	3 (20%)

*Note*: Data cutoff date.

Six patients were treated at dose level 1, six patients at dose level 2, and three patients at dose level 3. There were no dose‐limiting toxicities (DLTs) at dose level 1. One patient had a DLT on dose level 2, which was an infusion reaction to avelumab. Hence the dose level was expanded to six patients without further DLTs. There were two patients with DLTs at dose level 3 (respiratory failure in one patient and palmar‐plantar erythrodysesthesia as well as fatigue in the second patient). Per protocol, the maximum‐ tolerated dose (MTD) was dose level 2: avelumab 10 mg/kg every 2 weeks, axitinib 5 mg by mouth twice per day, and palbociclib 75 mg by mouth daily on days 8–28 of a 28‐day cycle (Table S1). However, due to multiple treatment interruptions and dose reductions occurring beyond the DLT window in dose level 2 (first 4 weeks of treatment), dose level 1 was expanded to six patients; based on the tolerable side effect profile of dose level 1 compared to dose level 2 (see Table 3, five of six patients at dose level 2 versus one of six patients at dose level 1 had ≥1 drug held during the first 60 days of treatment), the MTD (dose level 2) was declared by the Clinical Monitoring Committee to be above the recommended Phase II dose (RP2D). Thus, dose level 1—avelumab 10 mg/kg every 2 weeks, axitinib 3 mg by mouth twice per day, and palbociclib 75 mg po daily 7 days off/21 days on—is the RP2D.

**TABLE 3:** Detailed overview of 15 patients treated on SPRING trial

ID#Age/SexDose level	Histology	Prior therapy (no.)Prior checkpoint blockade (Y/N)	PDL1 + IHC (%)^a^	TMB^a^	Genomic results (vendor)^a^	Best response	PFS (weeks)^b^	Any drug held or reduced in first 60 days^c^	DLT (yes or no) comment
1 80/Flevel 1	Adenocarcinoma	2:Y	25%	Could not be determined	KRAS G12V, DNMT3A V675fs^b^38, MS could not be determined (FM)	SD	27	No	No
2 52/Mlevel 1	Adenocarcinoma	1:Y	0%	Intermediate, 6 muts/Mb	FGFR4 amplification, KRAS G12V, Myc amplification equivocal, PIK3CB amplification, C11orf30 (EMSY) amplification equivocal, CCND3 amplification, EPHB1 amplification, PRKCI amplification, TERC amplification, TP53 K120fs^b^1, TSHR A623S, VEGFA amplification, MS stable (FM)	PR	24	No	No
3 64/Flevel 1	Adenocarcinoma	0:N	60%	Not done	TP53 splice site 981_993 + 34del47, ERBB2 A775_G776insYVMA, ERRFI1 S138fs^b^8, ERRFI1 Q88^b^, MS not done (FM)	PR	181+	Yes, axitinib held	No
19 80/Flevel 1	Adenocarcinoma	1:Y	0%	Low, 4 Muts/Mb	No alterations, MS stable (FM)	SD	64	No	No
20 72/M level 1	Adenocarcinoma	1:N	0%	Low, 5 Muts/Mb	C17orf39 amplification, KMT2A (MLL) R1976Q, Myc amplification, RAD21 amplification, SMARCA4 R1189Q, STK11 K262, TP53 loss, MS stable (FM)	PD	8	No	No
21 61/M level 1	Squamous	1:Y	5%	Intermediate, 6 Muts/Mb	SOX2 amplification, CDKN2A D108H, EP300 Y1414C, FGF12 amplification, NFE2L2 D29H, NTRK3 E412K, POLE K733N—subclonal TP53 C238W, VHL truncation intron 1, MS stable (FM)	SD	15	No	No
5 69/Mlevel 2	Squamous	1:N	0%	Not done	P53, Ki67, RET.5p_NM_020975.4e6e7, MS not done (Histopath)	PD	7	Yes, course 2 delayed	No
4 63/Mlevel 2	Adenocarcinoma	2:Y	<1%	Not done	TP53 mutation, MS not done (Oncomine)	SD	29	Yes, palbociclib held	No
9 68/Flevel 2	Undifferentiated large cell carcinoma	2:Y	60–70%	Not done	No mutations, MS not done (Sheba Medical Center In house test: INFINITI®EGFR assay and Ion Torrent PGM system)	Not evaluable^e^	Not evaluable^e^	Yes, avelumab discontinued	Yes Avelumab infusion reaction
11 67/Mlevel 2	Adenocarcinoma	1:N	TPS <1%	Not done	No mutations, MS not done (Illumina TruSight)	SD	18	Yes, palbociclib held	No
13 72/Mlevel 2	Adenocarcinoma	1:N	0%	Not done	EGFR, ALK and ROS1 negative, MS not done (Unknown vendor)	PR	25	Yes, course 2 delayed	No
10 51/Mlevel 2	Adenocarcinoma	1:Y	70%	Not done	No mutations, MS not done (Illumina TruSight)	PR	14	No	No
15 76/Mlevel 3	Squamous	1:Y	Not done	Not done	Not done	SD	6	Yes, palbociclib reduced	Yes Respiratory failure^d^
14 51/Mlevel 3	Large cell neuroendocrine	2:Y	0%	Intermediate, 11 Muts/Mb	AR1D1A G328fs^b^35, STK11, LRPIB, KEAP, NFI, CCND1 amplification, KRAS amplification, FGF19 amplification, FGF3 amplification, FGF4 amplification (FM) MS stable (Caris)	SD	11	Yes, palbociclib and axitinib held	Yes Grade 3 fatigue and palmar‐plantar erythrodysesthesia
16 54/Flevel 3	Adenocarcinoma	2:Y	50%	Low, 4 Muts/Mb	FAT1 D3519fs^b^13, TET2 K542fs^b^19, TP53 TI55fs^b^26, MS Stable (FM)	SD	24	Yes, axitinib held	No

Abbreviations: DLT, dose‐limiting toxicity; F, female; FM, Foundation Medicine; IHC, immunohistochemistry; M, male; mb, megabase; MS, microsatellite; muts, mutations; N, no; PFS, progression‐free survival; TMB, tumor mutation burden; TPS, tumor proportion score; Y, yes.

^a^ Genomic results, TMB, and PDL1 may have been obtained as part of routine care; results were obtained prior to SPRING trial.

b “+” sign indicates ongoing PFS (data cutoff April 8, 2021 for PFS assessment).

c Drugs might be held or dose reduced because of Grade 2 side effects, in addition to Grade 3 or greater side effects.

d DLT was death due to respiratory failure possibly drug related.

e Not evaluable because patient did not receive full course of therapy; had infusion reaction to avelumab with first dose. Further, withdrew consent during course 2.

### 3.3 Responses and molecular/biologic findings

**FIGURE 1:**
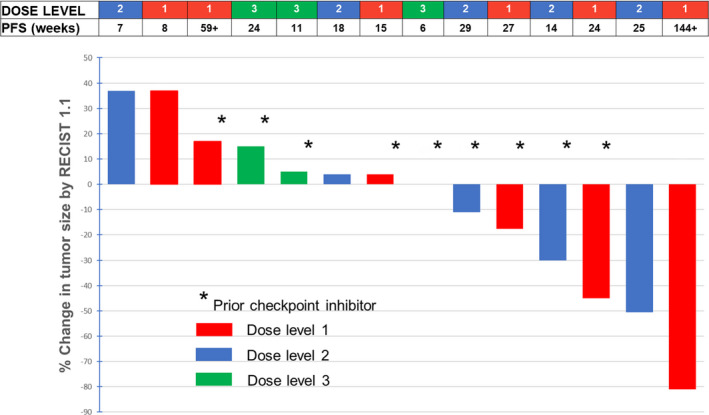
Waterfall plot of responses (*n* = 15 patients treated; (14 patients are shown; one patient [ID#9, Table 3]) was not evaluable because did not receive the full course of therapy due to infusion reaction to the first avelumab dose and withdrew consent during course 2; patient ID#15 had evaluable, but not measurable disease that was considered stable as best response). PFS, progression‐free survival; *Patient who received prior checkpoint inhibitor

**FIGURE 2:**
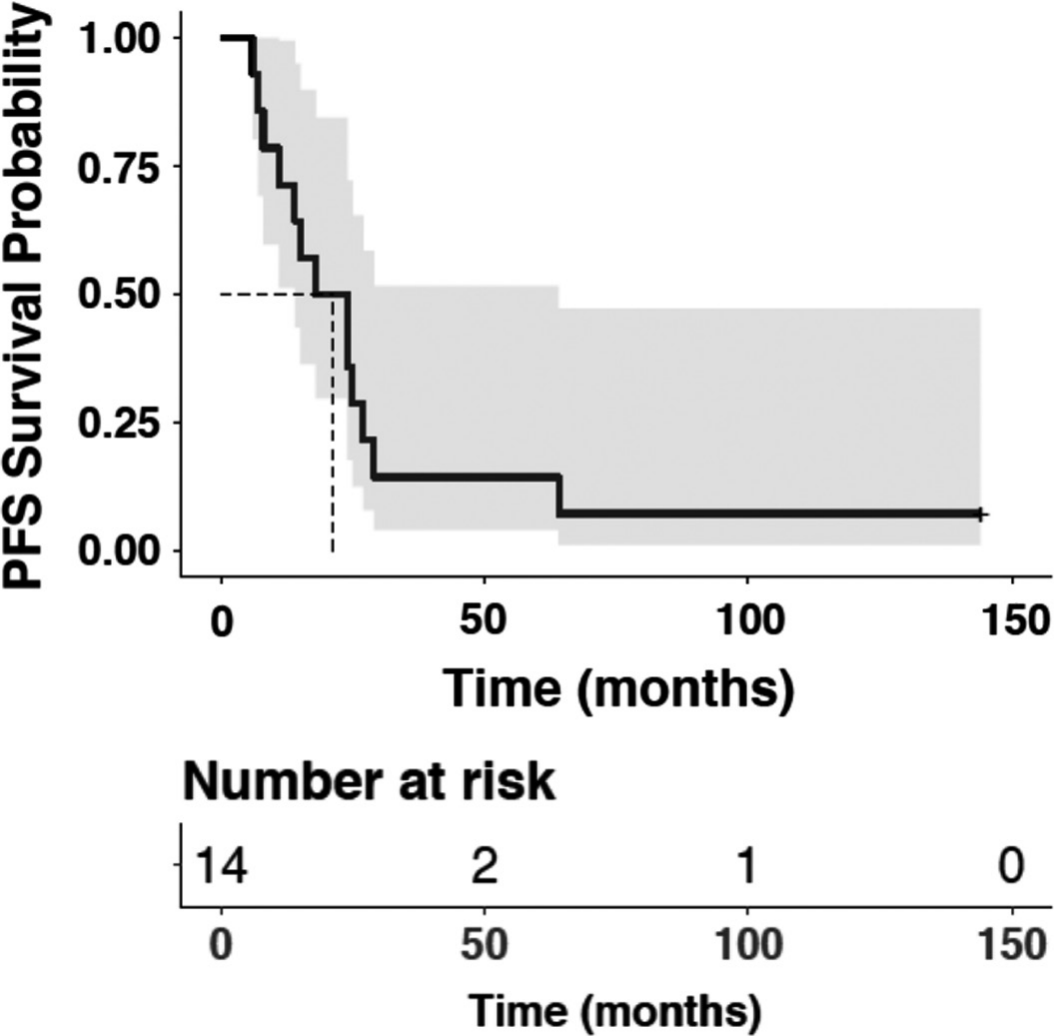
Kaplan–Meier curve of progression‐free survival of patients on study

Among the 15 patients treated, one patient was considered inevaluable for response because that patient did not receive a complete course of therapy (infusion reaction to avelumab on day 1) (Table 3). Four patients (27%) achieved a partial response (PR), including two PRs in patients who had previously experienced progression on pembrolizumab. Two of the PRs occurred in patients on dose level 1, indicating that this dose level (the RP2D) shows clinical activity (Figure 1). The PFS in the four patients with PRs was 14, 24, 25, and 144+ weeks (Figure 2 for Kaplan–Meier for PFS of patients on study). Four patients had stable disease (SD) that lasted ≥24 weeks: 24, 27, 29, and 64 weeks. Therefore, a total of eight of 15 patients (53%) achieved clinical benefit (SD ≥ 24 weeks and/or PR).

The patient with the longest response is a 64‐year‐old woman with no prior therapies in the metastatic setting, who had PD‐L1 positivity of 60% on IHC and an *ERBB2* exon 20 insertion alteration (Table S1); her tumors have shown >80% regression on imaging, with an ongoing PFS of more than 2.5 years (144+ weeks); it is suspected that she might have a complete remission because the residual disease in her lungs is small (but a biopsy has not been done) (Figure S1). The other three PRs were attained in patients with 0%, 0%, and 70% PD‐L1 positivity by IHC.^15^ Among the four patients achieving PR, two had available tumor mutation burden (TMB) and both were below 10 mutations/Mb.

## 4 DISCUSSION

Within the last decade, the treatment of advanced NSCLC has evolved dramatically. Treatment selection in this disease is influenced by molecular profiling as well as immunohistochemical evaluation for expression of PD‐L1^16^, ^17^ Although drugs targeting important molecular alterations have revolutionized outcomes for selected patients, many patients with NSCLC have complex tumors with oncogenic mutations and alterations leading to loss of function in tumor suppressor genes. The regimen in the current study addresses several important targets, including PD‐L1, VEGF, and CDK4/6 with avelumab, axitinib, and palbociclib, respectively. Previous preclinical and clinical studies have suggested a role for rational combination therapy with these particular agents individually or in combination in NSCLC and/or other cancers.^9^, ^10^, ^11^, ^18^, ^19^, ^20^

The SPRING trial explored the triple‐targeted therapy combination in the treatment of NSCLC. This Phase I study demonstrates the safety and tolerability of avelumab, axitinib, and palbociclib in advanced NSCLC without established oncogenic drivers. This tri‐therapy combination was well tolerated at the RP2D and showed encouraging activity in patients with advanced NSCLC at this dose level, with two of six patients experiencing a PR (including one ongoing at 144+ weeks) and an additional two patients experiencing prolonged stable disease (27 and 64 weeks).

Adverse events associated with the combination of avelumab, axitinib, and palbociclib were in line with previously described side effects of the individual drugs. The most common Grade 3 or higher adverse events were neutropenia, hypertension, and fatigue. The MTD was dose level 2, but because patients at dose level 2 often required dose reductions with ongoing therapy, dose level 1 (Table S1) was considered the RP2D (with most patients [five of six] at dose level 2 vs. one of the six patients at dose level 1 having had at least one or more drugs held during the first 60 days of treatment.)

Overall, 53% of patients (eight of 15) achieved clinical benefit (SD ≥ 24 weeks and/or PR) (includes four PRs lasting 14 to 125+ weeks) and four SD ≥ 6 months (lasting 24–64 weeks). Two of the PRs occurred in patients whose disease had previously progressed on pembrolizumab, suggesting that there may be synergy/additive effects between the avelumab and the other agents (axitinib and palbociclib) that serves to overcome immunotherapy resistance. Alternatively, it is possible that the antitumor effects were due to the targeted drugs, rather than the immunotherapy. Two of the six patients at dose level 1 (the RP2D) achieved a PR including the patient with the best overall response (~80% regression, ongoing at over 3 years) (Figure S1). No patient with clinical benefit had a known high tumor mutational burden, and two patients achieving PR had no expression of PD‐L1 on immunohistochemistry.

## 5 CONCLUSIONS

In conclusion, avelumab, axitinib, and palbociclib is generally well tolerated at dose level 1 and exhibits encouraging evidence of activity in patients with advanced NSCLC at that dose level, with four of six patients (66%) attaining SD ≥6 months/PR. The RP2D for the combination is avelumab 10 mg/kg IV every 2 weeks, axitinib 3 mg by mouth twice per day, and palbociclib 75 mg by mouth daily on days 7–28 of each 28‐day cycle. Responding patients included those with low TMB and no PD‐L1 expression on immunohistochemistry. Future study is warranted to further explore antitumor activity of this triplet combination of immunotherapy and targeted therapy to better identify biomarkers predictive of response.

## CONFLICT OF INTEREST

Dr. Benjamin Solomon discloses advisory board participation with AstraZeneca and Bayer. Dr. Ana Callejo receives advisory role and/or travel compensation from: Bristol‐Myers Squibb, F. Hoffman‐LaRoche, Pfizer, Boehringer Ingelheim, MSD Oncology, Kyowa Kirin, Celgene, Leo Pharma, Medscape, Kern Pharma. Dr. Jair Bar receives advisory board fee from Abbvie, AstraZeneca, Bayer, Bristol‐Myers Squibb, Boehringer Ingelheim, Causalis, Merck Sharp & Dohme, Novartis, Pfizer, Roche, Takeda; Research support (for the institution) from Abbvie, AstraZeneca, ImmuneAI, Merck Sharp & Dohme, Novartis, OncoHost, Roche, Takeda. Dr. Lyudmila Bazhenova has stock and other ownership interests in Epic Sciences; consulting or advisory role in Neuvogen, Janssen, Daichi Sankyo, Boehringer Ingelheim, Merck, Regeneron, Bristol‐Myers Squibb, Novartis; and receives research funding from BeyondSpring. Dr. Pierre Saintigny receives research grants from the following companies: AstraZeneca, Roche, Bristol‐Myers‐Squibb, Healthcare company Hitachi, Ose Immunotherapeutics, HTG Molecular Diagnostcs, Illumina, Cellenion, Vortex Biosceinces and Inivata. Dr. Vladimir Lazar, Catherine Bresson and Fanny Wunder are full time employees of Worldwide Innovative Network (WIN) Association—WIN Consortium. Worldwide Innovative Network (WIN) Association—WIN Consortium is the owner of the patent family entitled “Method for Selecting Personalized Tri‐Therapy for Cancer Treatment.” The inventor is Dr. Vladimir Lazar. Dr. J. Jack Lee receives honorarium from AstraZeneca for participating the AAZPIRE Education Program. Shai Magidi receives consultancy from Worldwide Innovative Network (WIN) Association—WIN Consortium. Dr. Amir Onn receives consulting fees from Roche Israel, MSD Israel, Boehringer Ingelheim and AstraZeneca. Dr. Brian Leyland Jones serves on the Board/ is an officer of NFCR, receives speaker's bureau fees from Puma, Genentech, Exelixis, Bayer. Dr. Richard L. Schilsky receives research grants in support of a clinical trial that he directs for the American Society of Clinical Oncology from the following companies: AstraZeneca, Bayer, Boehringer Ingelheim, Bristol‐Myers Squibb, Genentech, Lilly, Merck, Pfizer, Seattle Genetics. Dr. Schilsky serves as a consultant to: Brylogyx, Cellworks Group, Clarify Precision Oncology, EQRx, Scandion Oncology. Dr. Enriqueta Felip receives advisory board and/or speaker's bureau fee from Amgen, AstraZeneca, Bayer, Beigene, Boehringer Ingelheim, Bristol‐Myers Squibb, Eli Lilly, F. Hoffman‐LaRoche, Glaxi Smith Kline, Janssen, Medical Trends, Medscape, Merck Sharp & Dohme, Merck Serono, Peptomyc, Peervoice, Pfizer, Puma, Regeneron, Sanofi, Springer, Syneos Health, Takeda, Touch Medical; on the board of Grifols, Independent member. Receives research funding from Fundacion Merck Salud, Grant for Oncology Innovation (GOI) EMD Serono. Dr. Razelle Kurzrock receives research funding from Genentech, Merck Serono, Pfizer, Boehringer Ingelheim, TopAlliance, Takeda, Incyte, Debiopharm, Medimmune, Sequenom, Foundation Medicine, Konica Minolta, Grifols, Omniseq, and Guardant, as well as consultant and/or speaker fees and/or advisory board for X‐Biotech, Neomed, Pfizer, Actuate Therapeutics, Roche, Turning Point Therapeutics, TD2/Volastra, Bicara Therapeutics, Inc., has an equity interest in IDbyDNA and CureMatch Inc, serves on the Board of CureMatch and CureMetrix, and is a co‐founder of CureMatch. All other authors declare no potential conflict of interest.

## AUTHOR CONTRIBUTIONS

**Benjamin Solomon:** Investigation, resources, writing—original draft, review and editing, supervision. **Ana Callejo**: Investigation, resources, writing—review and editing. **Jair Bar**: Investigation, resources, writing—review and editing. **Guy Berchem**: Investigation, resources, writing—review and editing. **Lyudmila Bazhenova**: Investigation, resources, writing—review and editing. **Pierre Saintigny**: Writing—review and editing. **Fanny Wunder**: Data curation, project administration, visualization, writing—review and editing. **Jacques Raynaud**: Conceptualization, writing—review and editing. **Nicolas Girard**: Writing—review and editing. **J Jack Lee**: Conceptualization, formal analysis, writing—review and editing. **Raed Sulaiman**: Resources, validation, writing—review and editing. **Bruce Prouse**: Resources, validation, writing—review and editing. **Catherine Bresson**: Project administration, funding acquisition, writing—review and editing. **Hila Ventura**: Data curation, resources, software, writing—review and editing. **Shai Magidi**: Data curation, resources, software, formal analysis, methodology, writing—review and editing. **Eitan Rubin**: Resources, software, formal analysis, methodology, writing—review and editing. **Brandon Young**: Resources, validation, writing—review and editing. **Amir Onn**: Investigation, resources, writing—review and editing. **Brian Leyland‐Jones**: Investigation, resources, project administration, writing—review and editing. **Richard L. Schilsky**: Conceptualization, supervision, writing—review and editing. **Vladimir Lazar**: Conceptualization, resources, supervision, project administration, writing—review and editing. **Enriqueta Felip**: Investigation, resources, supervision, writing—review and editing. **Razelle Kurzrock**: Investigation, resources, supervision, project administration, writing—original draft.

## ETHICS APPROVAL AND CONSENT TO PARTICIPATE

All patients provided informed consent according to local institutional review board (IRB) guidelines: Avera Cancer Center, Sioux Falls, SD, USA: WIRB IRB 10/20/2017 tracking 20,172,298 WO number 1‐1038118‐1. Moores Cancer Center, UCSD, La Jolla, CA, USA: University of California San Diego, Human Research Protections Program 858–246‐4777 10/12/2017. Vall d'Hebron Institute of Oncology, Barcelona, Spain: Vall d'Hebron Hospital Comite de Etica de la Investigacion con Medicamentos (CEIM) 16/03/2018 act 331) 46228895E. Centre Hospitalier du Luxembourg, Luxembourg: Comite National d'Ethique (CNER) 15 Nov 2017 number 201707/01. Chaim Sheba Medical Center, Ramat Gan, Israel: The State of Israel Ministry of Health 20,174,800. The Chaim Sheba Medical Center Ethics Committee institutional Helsinki committee. 4637‐17_SMC and 4638‐17‐SMC. The study conforms to the Declaration of Helsinki, the Good Clinical Practice (GCP) guidelines of the International Council for Harmonization (ICH), US Codes of Federal Regulations for the Protection of Human Subjects, IND Application and Security and Privacy, Directive 2001/20/EC, Directive 2005/28/EC and EU General Data Protection Regulation (GDPR) 2016/679, as well as country‐specific regulations.

## Supporting information


Table S1

Figure S1

## Data Availability

Patient level data included in Table 3. Additional data available on request. The datasets used and/or analysed during the current study is available in Table 3. Additional data available upon request to the corresponding author on reasonable request.
