# Supplementary material for: A WIN Consortium phase I study exploring avelumab, palbociclib, and axitinib in advanced non‐small cell lung cancer
Source: Cancer Med. 2022 Mar 20;11(14):2790–800. doi: 10.1002/cam4.4635 (PMC9302335; doi:10.1002/cam4.4635)
Supplement: Supplementary file 1 — Table S1 [file CAM4-11-2790-s001.docx]

**Supporting Data**

**Supporting Table 1: Dose levels administered to patients as course 1 in SPRING trial^1,2^**

|  | **Dose Level 1** | **Dose Level 2** | **Dose Level 3** |
| --- | --- | --- | --- |
| **Avelumab** | 10 mg/kg every 2 weeks | 10 mg/kg every 2 weeks | 10 mg/kg every 2 weeks |
| **Axitinib** | 3 mg po bid | 5 mg po bid | 5 mg po bid |
| **Palbociclib** | 75 mg po qd 7 days OFF, 21 days ON | 75 mg po qd 7 days OFF, 21 days ON | 100 mg po qd 7 days OFF, 21 days ON |

^1^ In order to determine starting dose and dose-escalation plan, the following information was used. The Phase I of the dual combination avelumab and axitinib has been done and the recommended dose of the combination was avelumab (10 mg/kg IV every two weeks -1/+3 days) and axitinib (5 mg po bid). These doses are used in the study JAVELIN Renal 101, a randomized Phase III trial, referenced at US Clinical Trials website: <https://clinicaltrials.gov/ct2/show/NCT02684006?term=avelumab+and+axitinib&rank=1>

In order to add the third agent palbociclib, the dual drug combination dose was reduced and the third drug was added.

^2^Recommended Phase II dose (RP2D) was dose level 1; MTD was dose level 2

**Abbreviations**: kg = kilograms; mg= milligrams; MTD = maximum tolerated dose; po = by mouth; qd = daily
