# Supplementary figures and images for: A WIN Consortium phase I study exploring avelumab, palbociclib, and axitinib in advanced non‐small cell lung cancer
Source: Cancer Med. 2022 Mar 20;11(14):2790–800. doi: 10.1002/cam4.4635 (PMC9302335; doi:10.1002/cam4.4635)

**Supplemental Figure 1.**

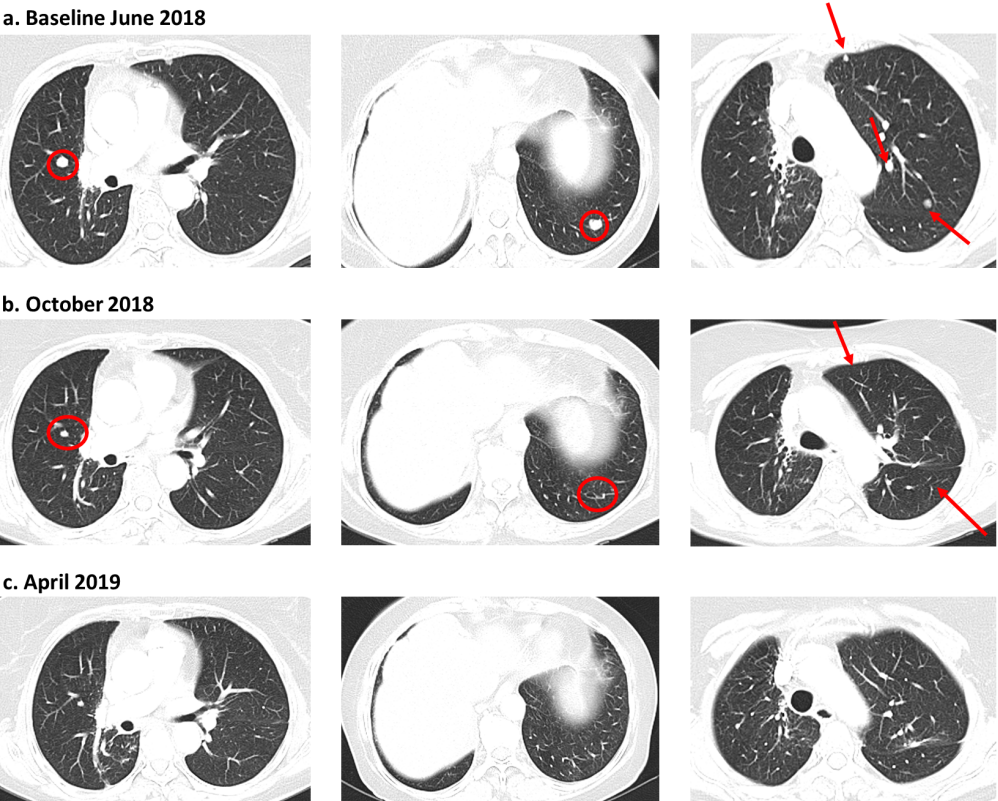

Supplement: Supplementary file 2 — Figure S1 [file CAM4-11-2790-s002.pdf]
